# Supplementary material for: Systematic empirical evaluation of individual base editing targets: Validating therapeutic targets in USH2A and comparison of methods
Source: Mol Ther. 2025 Jan 28;33(4):1466–84. doi: 10.1016/j.ymthe.2025.01.042 (PMC11997516; doi:10.1016/j.ymthe.2025.01.042)
Supplement: Document S1. Figures S1–S3 and Tables S1–S4 [file mmc1.pdf]

YMTHE, Volume *33*

Supplemental Information

Systematic empirical evaluation of individual base editing targets: Validating therapeutic targets in *USH2A* and comparison of methods

Yuki Tachida, Kannan V. Manian, Rossano Butcher, Jonathan M. Levy, Nachiket Pendse, Erin Hennessey, David R. Liu, Eric A. Pierce, Qin Liu, and Jason Comander

TG1

P1	C-m1	C-m2	Dmnt1	P2	C-m3	C-m4	P3	P4	C-m5	C-m6	C-m7	P5	C-m8	C-m9	P6	P7	A-m1	A-m2	A-m3	P8	A-m4	A-m5	P9	P10	A-m6	A-m7	A-m8	P11	A-m9	A-m 10	P12		

GGCGCGCCCCCAGTCACGACGTTGTAAAACGGCCTTTTCTTAAAGATGATCTCTTACCTTGGGAAAGGAGAGGTGTTCAA
TTCCTTGCACAGCTCACAGTTCCTTCCCGCATCAGGGAAAGGTTATGCATTATGGGGAGGCAAGCGCAGGCACTCGGGCT
GGAGCTGTTCGCGCTGGCATCTTGCTGGTCATCATCCTGCCTTTCAGGTGGGCTTGATGGCTTGTTCCCCGTAAGAAAAT
TAACAGGTTAAGTTGATTTTACGTCTCAGAAGCTCATATCCAAAGCAAAAGACAAGCAGGAACATCCTGTTCCTGTACGGC
ATGGTGTTATATGGAGGGGGCAAAGGTTTCTGTTGGCATTGGTGATTCCCGCTGTGGCAGAATGCCGTACTCCACATGCA
CATTGGTGGTCTCAATGTAACAGGAATATTTGGTATATGGTAACAGCGACACAACAAGCCAGCCATATAGACGCCTCTGCT
CCCAGGAGAGCTTCACTCTTTGATGACGGCCATGTCTGAAACCTTATAGGATATATGAGTCTACTATTACTCTCTGCAATTC
AGTTTCTTGAAGATACAATTGTCTTAAACCGGAGAAAACCTATACAATCAAATGgCCTGTGTGAAATTGTTATCCGCTGACT
GGTTCCAATTGACAAGCTCCAAGGGTTCAGTGGAGAGTCTGTAGTCATTAACACAAACTCTGGAAGCTGCTACTGGTGGC
ACAGCCAGCAACCATGCAAGCTTTCAGCTGATATGAATATCACATCCAACATCATTAAAGCTTCAGAGAAATTTAAATCCAA
AATTGCAAAATCTCGCCGGATCCTAACTAAAATAATTCGCTATGAAGTGATTCACAGATGCTTCGAGGGAAAAGCTTGGTT
GCTGTCCACCGAAAATGGGGCTCAGTACTGAGGCACTGTGGGGAGAAAGAGTCAAGTAACAACCGCGACATATCGGTGG
TCATCATGCATCAAACCTTCAGGCACTTTTGTTCAAAAGCTGGCCTTAATGCCTGGTAAGTTCTCCAGCCTTGGGAAAAGA
CCTCATGACTCAGTCAAGGATATTGAAGCACACACTAGCTGTGCAACCTCCATTCATGCAGGCTACAAGGGAGAAGTTATA
CCTTATGCAGTTGCTCTCCCATGTTCTCTGGCAGGGCCTCCATTATCCAGGCAGATTTTGACACACTAGCatttgcagGTTTAG
AGCAGTTTGTCAGAAGAATGCAAGATTTTCGATTATAGGATGTCAGAATGCCATTTGCACGCGT

TG2

P3	C-m10	C-m11	Dmnt1	A-m11	A-m12	P10	P4	A-m13	A-m14	A-,m15	A-m16	P6	P7	A-m17	A-m18	A-m19	A-m20	A-m21	P11	P9	A-m22	A-m23	A-m24	P12			

GGCGCGCCCTGTTCCTGTACGGCATGGATTCTGCAATCCTCACTCTGGGCAGCGTGAGTGCAAAAAAGAAGCCAAAGGC
GGAGGAACTACAGGTCCAGGTTCTCTAAAGTAAAATAAATCCAGAAAGTCGGGGAGGCAAGCGCAGGCACTCGGGCTGG
AGCTGTTCGCGCTGGCATCTTGAGAGGATGGGCTTCAGGATTCAACCATGACACTCTATTATCAGCTGTGTCTGCCGAAG
GATCTGCACCAACTTGTCAGAAAAAGTAATCCTTGCACAGCTCACATATCGGTGGTCATCATGCCTGGTCATCATCCTGCC
TTTTCAACATTGGGCTTGCAGATGCACTACCCTGTCTTAGCATTACAGACAGTCATTTCTTTCTTACCTGGTTGACACTAATT
ACACCTTCTTCCTTGACGATTAAGCCAACCACTGCTCTGAAAAACTCAACTTTCCTCAGATGTGGTTTCTTTATCCTTCCTG
AGGATGGTATAACTTCACGGGAGCCCTCCCAGAAAGACTCCTCCTGTGTGAAATTGTTATCCGCTGACTGGTTCCAATTGA
CAAGCCCCTTACTATTTGCTAGGTTTCGGTAGTTGCATGAAGGATGTTAAATTTACACAGGTGGCTGCACCCCAGCAGGTC
ATGAGGGTCTTGTGGTAACTTCTACCACAATCTGTCTGCCACAGCACTTCTAGCCATGGCCATCATGAAGCCTCCCAAATC
TGGTTTCCTGAGGTGGAGTACAGCATTCTGCCACAGCAGGAATCACCTTTGCTGGTGGAGCATCCTCCACTCATGCAGGC
TTGGAGTGCATAGCTATAGGAGAGCAACTGCATAAGGAGTCAAGTAACAACCGCGACCCAGGGTGGTGCACGCTTGAATT
CATATTTCATACCTTCAGGACATAAGGAGAGGTATCATATTGGATCAACGGCATCTTAACACTTCCTTCGTCAGTCGTTGAC
GATTAGGCACACACAGGCACTAGCCACTGATTGGGTCACAAATGGTCGTCTGGGCTACATTGAAGCCGTGGCACCAGTTG
TGGTAGGTGATTCTTCGAGGATGTCAGAATGCCATTTGCACGCGT

Yellow: restriction enzyme site
Green: forward primer sequence
Light blue: reverse primer sequence

Figure S1. Transgene sequence with tandem USH2A mutation target sequences and universal primers (forward, green; reverse, blue) and restriction enzyme sites (yellow)

Figure S2

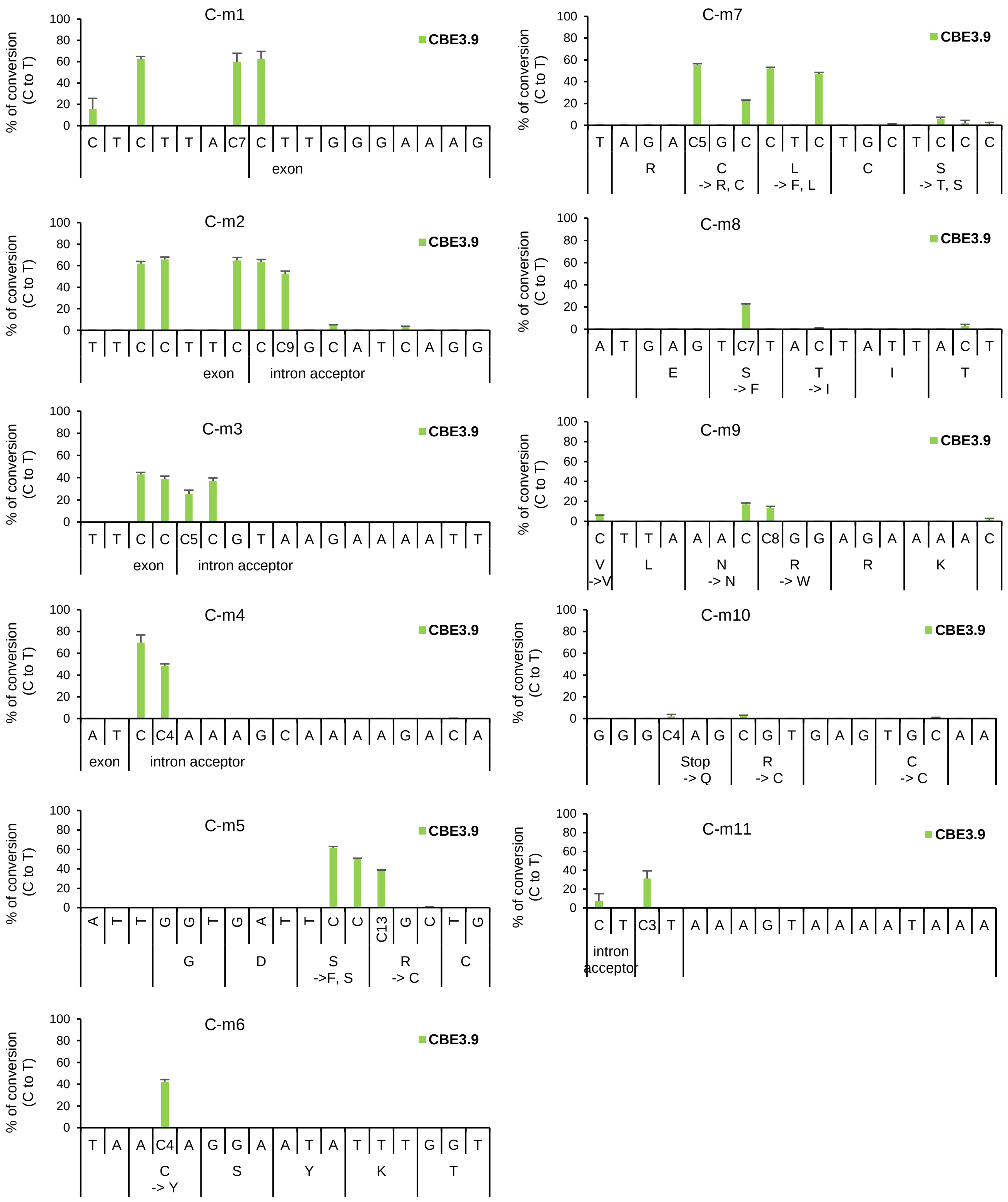

Figure S2. CBE editing efficiencies are shown for each basepair within the edited region. The target “C” basepair is numbered. Amino acid changes that take place with C-to-T editing are shown as indicated

Figure S3A

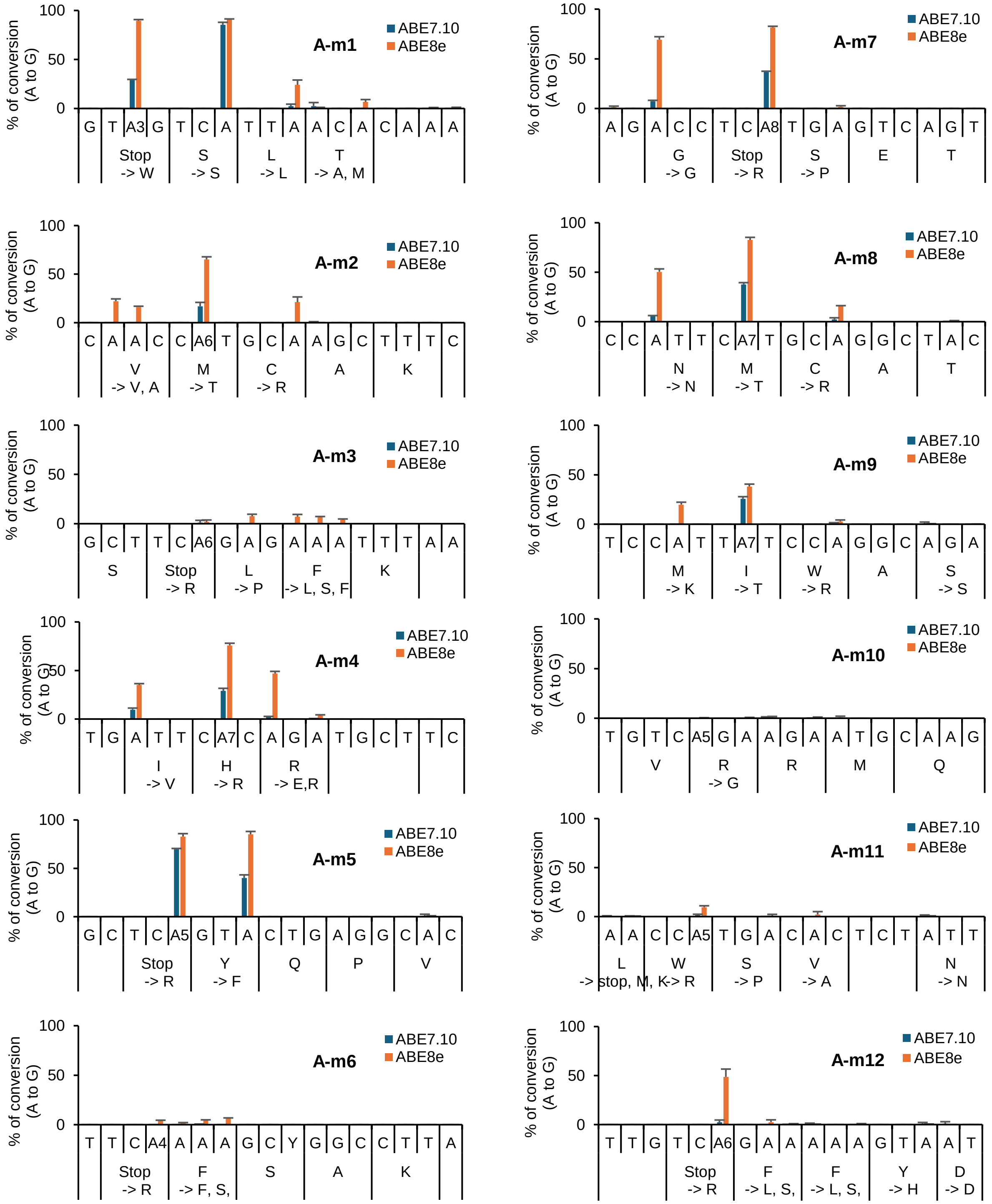

% of conversion (A to G)
ABE7.10
ABE8e
A-m1
G T A3 G T C A T T A A C A C A A A
Stop -> W
S -> S
L -> L
T -> A, M
A-m2
C A A C C A6 T G C A A G C T T T C
V -> V, A
M -> T
C -> R
A
K
A-m3
G C T T C A6 G A G A A A T T T A A
S
Stop -> R
L -> P
F -> L, S, F
K
A-m4
T G A T T C A7 C A G A T G C T T C
I -> V
H -> R
R -> E,R
A-m5
G C T C A5 G T A C T G A G G C A C
Stop -> R
Y -> F
Q
P
V
A-m6
T T C A4 A A A G C Y G G C C T T A
Stop -> R
F -> F, S,
S
A
K
A-m7
A G A C C T C A8 T G A G T C A G T
G -> G
Stop -> R
S -> P
E
T
A-m8
C C A T T C A7 T G C A G G C T A C
N -> N
M -> T
C -> R
A
T
A-m9
T C C A T T A7 T C C A G G C A G A
M -> K
I -> T
W -> R
A
S -> S
A-m10
T G T C A5 G A A G A A T G C A A G
V
R -> G
R
M
Q
A-m11
A A C C A5 T G A C A C T C T A T T
L
W
-> stop, M, K-> R
S -> P
V -> A
N -> N
A-m12
T T G T C A6 G A A A A A G T A A T
Stop -> R
F -> L, S,
F -> L, S,
Y -> H
D -> D

Figure S3B

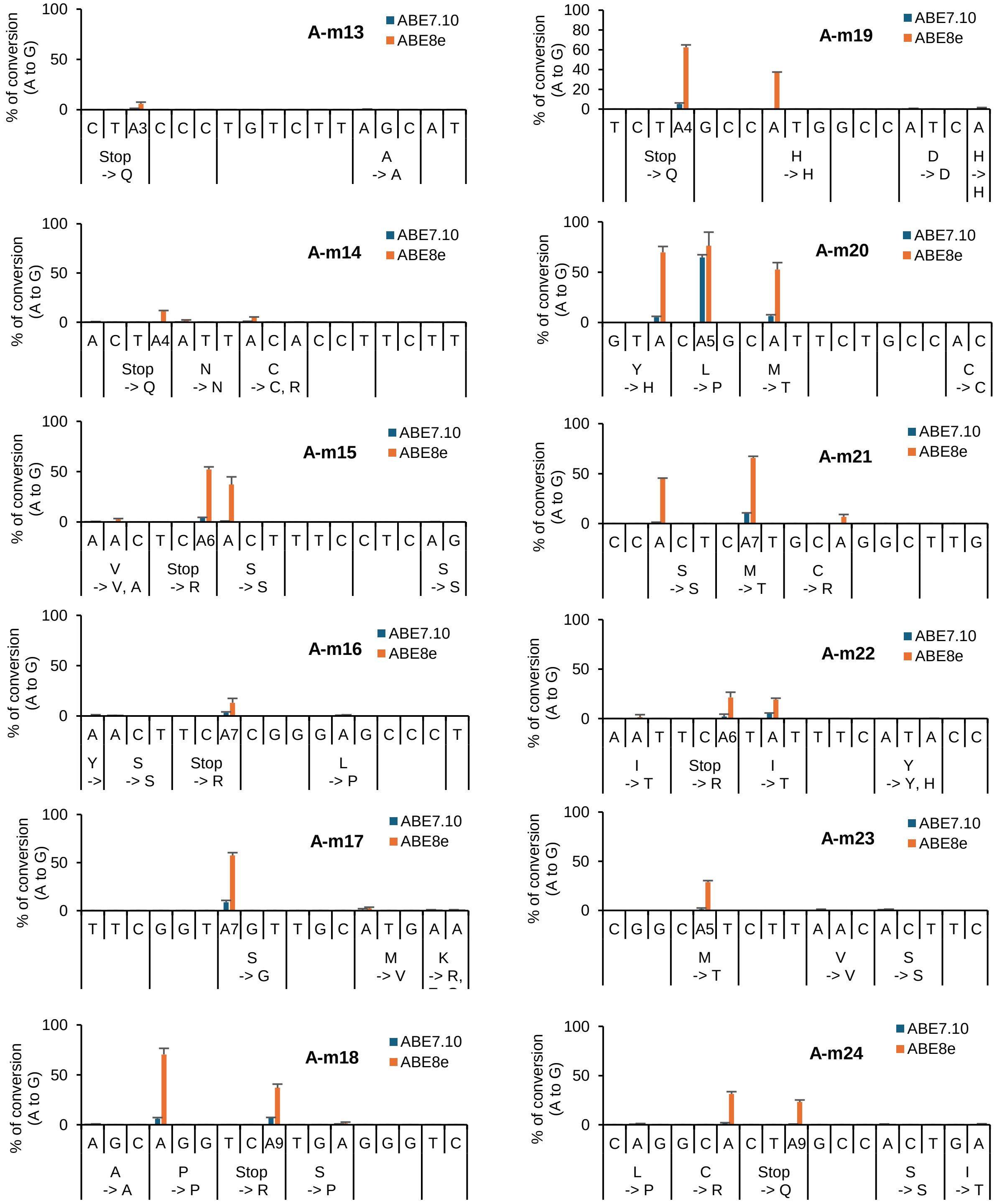

Figure S3A and S3B. ABE editing efficiencies are shown for each basepair within the edited region. The target "A" basepair is numbered. Amino acid changes that take place with A-to-G editing are shown as indicated

Table S1. Detailed descriptions of the predicted off-target editing sites for C-m6 and A-m1, as quantified in Figure 5

Target mutation	Chr.	Locus Description	HGVS format	Off target sequence	Mismatch count	Mismatch (*) position	Mit off-target score (CRISPOR)
C-m6	chr8	intergenic:CTD-2023J5.1-SGCZ	NC_000008.11:g.15132411 G>A	GAACAGGAAAATTTGGTATAAGG	2	*........*.........	7.414831
C-m6	chr20	intron:RALGAPA2	NC_000020.11(RALGAPA2_v001):n.486+3700C>T	AACCAGGGATATTTGGTATAAGG	3	*.*....*...........	2.507898
C-m6	chr2	intron:LRP1B	NC_000002.12(LRP1B_v002):c.12752+264G>A	ACTCAGGAATATTTGGTATAGGG	3	***................	2.287424
C-m6	chr6	intron:KIF6	NC_000006.12(KIF6_v002):c.1754+3517C>T	TTAAAGGACTATTTGGTATAGGG	3	.*.*....*..........	1.554083
C-m6	chrX	intergenic:RP3-326L13.3-RP1-223D17.1	NC_0000023.11:g.83515127 C>T	TATCAGGAGCATTTGGTATAAGG	3	..*.....**.........	1.411272
C-m6	chr5	intron:CTC-340A15.2	NC_000005.10:g.165104019 G>A	TAACAGGTATATTTGTTATAAGG	2	.......*.......*....	1.296825
C-m6	chr4	intergenic:RNA5SP168-LRBA	NC_000004.12:g.150971524 C>T	CAAAAGGAATATTTGGCATAAGG	3	*..*...........*...	1.290123
C-m6	chr13	intron:TDRD3	NC_000013.11(TDRD3_v003):c.217-525C>T	AAACAGGAAAATTTAGTATAAGG	3	*........*....*.....	0.777733
C-m6	chr3	intron:CACNA2D3	NC_000003.12(CACNA2D3_v001):n.1518+1271G>A	TAACAGGAATACTTTGTATATGG	2	..........*..*.....	0.754598
C-m6	chr7	intergenic:RP11-548K12.12-RP11-715L17.1	NC_000007.13:g.61770663 G>A	TAACAGGAATATATGTTATATGG	2	...........*..*....	0.380939
A-m1	chr5	intergenic:ATP10B-RP11-109J4.1	NC_000005.9:g.160332230 T>C	GTAGTCAAGAACACAAACTCTGG	2	.......**..........	3.189286
A-m1	chr11	intron:SOX6	NC_000011.9(SOX6_v001):c.1251+1418T>C	TTATTCATAAACACAAACTCTGG	3	*..*....*..........	1.632771
A-m1	chr1	intergenic:RP13-614K11.1-RP11-374C13.1	NC_000001.11(LOC105376673_v001):n.138T>C	GTGGTCATCTACACAAACTCTGG	3	..*.....**.........	1.411272
A-m1	chr10	intergenic:ATRNL1-RP11-169K19.1	NC_000010.11(ATRNL1_v001):n.*599259A>G	GAAGTCAATAAAACAAACTCTGG	3	.*.....*...*.......	1.384889
A-m1	chr2	intergenic:AC010967.2-SCARNA16	NC_000002.12:g.53254147 T>C	GAAGTTAGAAACACAAACTCTGG	4	.*...*.**..........	0.504558
A-m1	chr2	intergenic:AC016716.1-AC016670.1	NC_000002.12:g.80229784 A>G	TTACTCATTAACATAAACTCAGG	3	*..*.........*.....	0.443036
A-m1	chr11	intergenic:OPCML-OPCML-IT1	NC_000011.9(OPCML_v001):c.61+255482A>G	GAAGTTATAAAAACAAACTCGGG	4	.*...*..*..*.......	0.260206
A-m1	chr5	intergenic:RP11-284A20.2-RP11-284A20.1	NC_000005.9:g.124245586 T>C	GTAATCATTAGCATAAACTCAGG	3	...*......*..*......	0.232771
A-m1	chr21	intron:NCAM2	NC_000021.9(NCAM2_v001):c.55+89989T>C	GAAATCATAAACACAAAATCTGG	4	.*.*....*........*..	0.189614
A-m1	chr7	intron:CDK14	NC_000007.13(CDK14_v001):c.*29-38778A>G	GTTATCATTAACATAAATTCAGG	4	..**.........*...*..	0.045592

Table S2. Oligonucleotide sequence for cloning of sgRNA

**Primer sequence for cloning of guide RNAs**				
**Target site name**	**sgRNA (top)**	**sgRNA (bottom)**	**Type of Cas9**	**PAM**
C-m1	CACCGCTCTTACCTTGGGAAAGGAG	AAACCTCCTTTCCCAAGGTAAGAG	S.pyogenes	NGG
C-m2	CACCGTTCCTTCCCGCATCAGGGAA	AAACTTCCCTGATGCGGGAAGGAAC	S.pyogenes	NGG
C-m3	CACCGTTCCCCGTAAGAAAATTAAC	AAACGTTAATTTTCTTACGGGGAAC	S.pyogenes	NGG
C-m4	CACCGATCCAAAGCAAAAGACAAGC	AAACGCTTGTCTTTTGCTTTGGATC	S.pyogenes	NGG
C-m5	CACCGATTGGTGATTCCCGCTGTGG	AAACCCACAGCGGGAATCACCAATC	S.aureus	NNGAAT
C-m6	CACCGTAACAGGAATATTTGGTATA	AAACTATACCAAATATTCCTGTTAC	S.pyogenes	NGG
C-m7	CACCGTAGACGCCTCTGCTCCCAGG	AAACCCTGGGAGCAGAGGCGTCTAC	S.pyogenes	NGA
C-m8	CACCGATGAGTCTACTATTACTCTC	AAACGAGAGTAATAGTAGACTCATC	S.aureus	NNNAAT
C-m9	CACCGCTTAAACCGGAGAAAACCTA	AAACTAGGTTTTCTCCGGTTTAAGC	S.aureus	NNNAAT
A-m1	CACCGTAGTCATTAACACAAACTC	AAACGAGTTTGTGTTAATGACTAC	S.pyogenes	NGG
A-m2	CACCGCAACCGTGCAAGCTTTCAGC	AAACGCTGAAAGCTTGCACGGTTGC	S.pyogenes	NGA
A-m3	CACCGCTTCAGAGAAATTTAAATC	AAACGATTTAAATTTCTCTGAAGC	S.aureus	NNNAAT
A-m4	CACCGTGATTCACAGATGCTTCGAG	AAACCTCGAAGCATCTGTGAATCAC	S.pyogenes	NGA
A-m5	CACCGCTCAGTACTGAGGCACTGT	AAACACAGTGCCTCAGTACTGAGC	S.pyogenes	NGG
A-m6	CACCGTTCAAAAGCTGGCCTTAATG	AAACCATTAAGGCCAGCTTTTGAAC	S.aureus	NNNGGT
A-m7	CACCGAGACCTCATGACTCAGTCAA	AAACTTGACTGAGTCATGAGGTCTC	S.pyogenes	NGA
A-m8	CACCGCCATTCATGCAGGCTACAAG	AAACCTTGTAGCCTGCATGAATGGC	S.pyogenes	NGA
A-m9	CACCGTCCATTATCCAGGCAGATTT	AAACAAATCTGCCTGGATAATGGAC	S.pyogenes	NGA
A-m10	CACCGTGTCAGAAGAATGCAAGATT	AAACAATCTTGCATTCTTCTGACAC	S.aureus	NNNGAT
Dnmt1	CACCGCGGGCTGGAGCTGTTCGCG C	AAACGCGCGAACAGCTCCAGCCCGC	S.pyogenes	NGG
C-m10	CACCGGGCAGCGTGAGTGCAAAAA	AAACTTTTTGCACTCACGCTGCCC	S.pyogenes	NGA
C-m11	CACCGCTCTAAAGTAAAATAAATCC	AAACGGATTTATTTTACTTTAGAGC	S.pyogenes	NGA
A-m11	CACCGAACCATGACACTCTATTATC	AAACGATAATAGAGTGTCATGGTTC	S.pyogenes	NGC
A-m12	CACCGTTGTCAGAAAAAGTAATCCT	AAACAGGATTACTTTTTCTGACAAC	S.pyogenes	NGC
A-m13	CACCGCTACCCTGTCTTAGCATTAC	AAACGTAATGCTAAGACAGGGTAGC	S.pyogenes	NGA
A-m14	CACCGACTAATTACACCTTCTTCCT	AAACAGGAAGAAGGTGTAATTAGTC	S.pyogenes	NGA
A-m15	CACCGAACTCAACTTTCCTCAGATG	AAACCATCTGAGGAAAGTTGAGTTC	S.pyogenes	NGG
A-m16	CACCGAACTTCACGGGAGCCCTCCC	AAACGGGAGGGCTCCCGTGAAGTTC	S.pyogenes	NGA
A-m17	CACCGTTCGGTAGTTGCATGAAGGA	AAACTCCTTCATGCAACTACCGAAC	S.pyogenes	NGT
A-m18	CACCGAGCAGGTCATGAGGGTCTTG	AAACCAAGACCCTCATGACCTGCTC	S.pyogenes	NGG
A-m19	CACCGTCTAGCCATGGCCATCATGA	AAACTCATGATGGCCATGGCTAGAC	S.pyogenes	NGC
A-m20	CACCGTACAGCATTCTGCCACAGC	AAACGCTGTGGCAGAATGCTGTAC	S.pyogenes	NGG
A-m21	CACCGCCACTCATGCAGGCTTGGAG	AAACCTCCAAGCCTGCATGAGTGGC	S.pyogenes	NGC
A-m22	CACCGAATTCATATTTCATACCTTC	AAACGAAGGTATGAAATATGAATTC	S.pyogenes	NGG
A-m23	CACCGCGGCATCTTAACACTTCCTT	AAACAAGGAAGTGTTAAGATGCCGC	S.pyogenes	NGT
A-m24	CACCGCAGGCACTAGCCACTGATTG	AAACCAATCAGTGGCTAGTGCCTGC	S.pyogenes	NGT
**Primer sequences for PCR**				
**Target site name**	**Forward (5'->3')**	**Reverse (5'->3')**		
M13	CCCAGTCACGACGTTGTAAAACG	AGCGGATAACAATTTCACACAGG		
AOX	GACTGGTTCCAATTGACAAGC	GCAAATGGCATTCTGACATCC		
Luc	AGTCAAGTAACAACCGCGA	CCTTATGCAGTTGCTCTCC		
Bglo	CTGGTCATCATCCTGCCTTT	TTTGCCCCGCTCCATATAACA		
CMV-F	AAATGGGCGGTAGGCGTG			
XL39-F	ATTAGGACAAGGCTGGTGGG			
tdTomato	CTGTTCCTGTACGGCATGG	TCTTTGATGACGGCCATGT		
GW	AATCTCGCCGGATCCTAACT	GCATGATGACCACCGATATG		
PuroDNR-F	GGTCCACTAGTGCTGTCCTG			
AAVS-1	AGGTTCCGTCTTCCTCCACT	CTTGCCAAGGACTCAAACCC		
Ms Genotyping	GCAGACGTCTAGCACATTCCT	ACCGGTCCCAAATACAGCTC		
mUSH2A	GCACTCTGCGGCCTTTTACA	CATGGTACTGGGAGATGAGGC		

Table S3. Primer sequences for off-target sequence analysis

HGVS format	Forward primer	Reverse primer	Product bp
NC_000008.11:g.15132411G>A	GTCCCACATTCCTCAGACCC	TCCACTGGTTTTCATGCATGC	251
NC_000020.11(RALGAPA2_v001):n.486+3700C>T	GGCACAGCACTGTACACTGA	CAGCAAGATGACCAGGGGAG	395
NC_000002.12(LRP1B_v002):c.12752+264G>A	GACAGGTCTTTTGCTCCTCCT	AGTGTGTAAGGTGCCATGGG	493
NC_000006.12(KIF6_v002):c.1754+3517C>T	CGGGTTACTGGAAGGGCATT	CCCATCTTCCTTGTGGCCAT	447
NC_0000023.11:g.83515127C>T	TGGTGCAGCATTTCCCAAAC	CCCGCCTTGGAAGCCATATA	335
NC_000005.10:g.165104019G>A	GCACAGGCATTTCAAACAGA	CTGTCTTGCTCCTGCCATAT	399
NC_000004.12:g.150971524C>T	TCATCCCAGCCACCTTCTCT	TTTCCCTTCCTCTTTGGGCC	335
NC_000013.11(TDRD3_v003):c.217-525C>T	TGTGAGAAATTGCTTTTGCCAGA	TCAGGCCATTCCCCTAAGGA	462
NC_000003.12(CACNA2D3_v001):n.1518+1271G>A	TGGACAACCAGAGGCCTAGA	TTTCTACGGCTGATGGCACC	425
NC_000007.13:g.61770663G>A	TGCTTCTTTCTTGACTGCTTGT	ACAAGGAAAATTCAAGCTCGACC	415
NC_000002.12:g.53254147T>C	ACAGGGTTCACAATTCCAGCT	AGGCATGGGCAGCTCTTAAG	581
NC_000011.9(OPCML_v001):c.61+255482A>G	GGAGAAAAGGAAGAGGGGCC	CAAAGTCGTTGGGGAAGGGT	490
NC_000001.11(LOC105376673_v001):n.138T>C	CCTGGTACCTGATCCAACACC	TTTACCTTTGGTTGAATTCTTGGT	748
NC_000010.11(ATRNL1_v001):n.*599259A>G	TCAAGGCAGGCTGTGTCAAA	GCTTCAATTTCTACATTTGACGTCC	547
NC_000005.9:g.124245586T>C	GAAATTCTTCACTGCAGCTCCT	GCCCTCTCCTTCCCAGAGATA	450
NC_000021.9:g.21088607T>C	TCACTTTAGGGCGAGCATCA	TCTCTTCTTAAGACTGGAAAGGCT	490
NC_000011.9(SOX6_v001):c.1251+1418T>C	AGCTGGCTCACAAAGATGGA	AGGGAAACAGCTGAGACCAA	597
NC_000007.13(CDK14_v001):c.*29-38778A>G	TGGAAGAGGGTGCTATAACCT	AGCATCATGCCTGGCAAGTA	573
NC_000005.9:g.160332230T>C	TGTGTAAAGTTTAAACAAACTGGA	TGTTACATGCTACAGTTGGGT	755
NC_000002.12:g.80229784A>G	TACCTGAGGAATCAGATGGG	AGGAGTGTCTTTTGCTGTTT	755

Table S4. Primer sequences for cloning of USH2A mutant plasmid vector**.**

Primer name	5' -> 3'	PCR product (bp)
A-m1_5p_F	TGTCAGATACCATATGCTGCTCAGCTTCCAGTGGAGAGTCTAAAG	1882
A-m1_5p_R	GTTAATGACTACAGACTCTCCACTGAAC	
A-m1_3p_F	GAGAGTCTGTAGTCATTAACACAAACTCTGG	687
A-m1_3p_R	GTTAACAGGCTCAGACCAGCTCAGCTCAACACTGGTGGACTTC	
A-m7_5p_F	CAGATCCGCTAGAGATCCGCGGCCGCCACCATGAATTGCC	139
A-m7_5p_R	AAAGACCTCATGACTCAGTCAAGGATATTGAAGC	
A-m7_3p_R	GACTGAGTCATGAGGTCTTTTCCCAAGGCTGGAG	857
A-m7_3p_R	GTGCCAAAGGGTGGACCCGCGGGTGGCTGCCAGGGCAACG	
A-m12_5p_F	CAGATCCGCTAGAGATCCGCGGCCGCCACCATGAATTGCCCAGTTC	1915
A-m12_5p_R	CAACTTGTCAGAAAAAGTAATCCTTGCACAGC	
A-m12_3p_F	TTACTTTTTCTGACAAGTTGGTGCAGATC	156
A-m12_3p_R	TTGCACTGCCTGCCAGACACGTGTCTCTTACAATTACACTGTCCTC	
A-m15_5p_F	AGTGTAATTGTAAGAGACACGTGTCTGGCAGGCAGTGCAATCAG	1912
A-m15_5p_R	TGAAAAACTCAACTTTCCTCAGATGTGGTTTCTTTAG	
A-m15_3p_F	GAGGAAAGTTGAGTTTTTCAGAGCAGTG	1308
A-m15_3p_R	AAGTTCCAGGAACCCTGCTCCTAGGAACTGAGCTCCCTCATTTAATG	
